# Prognostic role of euthyroid sick syndrome in MIS-C: results from a single-center observational study

**DOI:** 10.3389/fped.2023.1217151

**Published:** 2023-08-10

**Authors:** Michele Fastiggi, Alessandra Meneghel, Joaquin Gutierrez de Rubalcava Doblas, Fabio Vittadello, Francesca Tirelli, Francesco Zulian, Giorgia Martini

**Affiliations:** ^1^ Pediatric Rheumatology Unit, Department of Woman and Child Health, University of Padova, Padova, Italy; ^2^ Pediatric Diabetes and Pediatric Metabolism Unit, Department of Woman and Child Health, University of Padova, Padova, Italy

**Keywords:** children, COVID-19, euthyroid sick syndrome, multisystem inflammatory syndrome, SARS-CoV2

## Abstract

**Background:**

Euthyroid sick syndrome (ESS) is characterized by low serum levels of free triiodothyronine (fT3) with normal or low levels of thyroid stimulating hormone (TSH) and free thyroxine (fT4) and is reported in different acute clinical situations, such as sepsis, diabetic ketoacidosis and after cardiac surgery. Our aim was to evaluate the predicting role of ESS for disease severity in patients with Multisystem Inflammatory Syndrome in children (MIS-C).

**Methods:**

A single-centre observational study on consecutive patients with MIS-C. Before treatment clinical, and laboratory data were collected and, in a subset of patients, thyroid function tests were repeated 4 weeks later. Variables distribution was analyzed by Mann-Whitney *U*-test and correlations between different parameters were calculated by Spearman's Rho coefficient.

**Results:**

Forty-two patients were included and 36 (85.7%) presented ESS. fT3 values were significantly lower in patients requiring intensive care, a strong direct correlation was shown between fT3 and Hb, platelet count and ejection fraction values. A significant inverse correlation was retrieved between fT3 levels and C-reactive protein, brain natriuretic peptide, IL-2 soluble receptor and S-100 protein. Subjects with severe myocardial depression (EF < 45%) had lower fT3 values than subjects with higher EF. The thyroid function tests spontaneously normalized in all subjects who repeated measurement 4 weeks after admission.

**Conclusion:**

ESS is a frequent and transient condition in acute phase of MIS-C. A severe reduction of fT3 must be considered as important prognostic factor for severe disease course, with subsequent relevant clinical impact in the management of these patients.

## Introduction

From May 2020, after the first reported cases of “Kawasaki-like disease” (1), a novel multisystem inflammatory syndrome in children (MIS-C) related to COVID19 infection was identified. It was defined by signs of systemic inflammation (fever, elevated inflammatory markers) and at least two organ dysfunctions in an individual aged <21 years, with current infection or exposed within 4 weeks to SARS-CoV2 (2). The clinical manifestations of MIS-C are very heterogeneous but cardiovascular and gastrointestinal systems appear to be the most affected (3). Few studies have explored possible manifestations affecting the endocrine system during this iperinflammatory disease related to SARS-CoV2.

Recently, an alteration of the thyroid axis known as *euthyroid sick syndrome* (ESS) has been reported in patients with MIS-C (4). This condition, described in children with severe clinical situations such as sepsis (5), diabetic ketoacidosis (6) or after cardiac surgery (7), is characterized by decreased levels of triiodothyronine (fT3), increased conversion of thyroxine (fT4) to the biologically inactive form of reverse T3 (rT3) without a compensatory rise in of thyroid-stimulating hormone (TSH) (8). The result is an overall reduction in the bioavailability of active fT3, thus miming central hypothyroidism (9).

ESS appears as an adaptive mechanism to reduce the energy expenditure of the organism. For this reason, there is no agreement to correct this condition although the severity of ESS is associated with poor outcome in some studies (10).

In this study, our aim was to collect and analyze the thyroid profile together with other laboratory and instrumental data in order to evaluate the possible prognostic role of ESS within a cohort of patients affected by MIS-C.

## Patients and methods

### Patients

A single-center observational study was conducted at the Pediatric Rheumatology Unit of the Department of Woman and Child Health of Padova between 30 November 2020 and 30 September 2022. Consecutive patients admitted for MIS-C, according to classification criteria proposed by the Center for Disease Control and Prevention (CDC), were included (3). Children with known thyroid, hypothalamic and pituitary disease or undergoing medical therapy with glucocorticoids or inotropes in the 48 h prior to admission were excluded. This study was approved by the Ethical Committees of Padova University Hospital (n. 338n/AO/23). Due to the nature of the study (observational cross-sectional), no informed consent was required from the patients and their caregivers.

### Clinical data collection

Laboratory tests were performed for each patient upon admission and before any treatment. More precisely, collected data were: complete blood count (CBC), C-Reactive Protein (CRP), Erythrosedimentation Rate (ESR), ferritin, cytokines and biomarkers profile (interleukins IL-1α, IL-1β, IL-6, soluble IL2 receptor, IL2r, tumor necrosis factor α, TNF α, and S-100 protein), organ involvement indices such as glutamic-pyruvic transaminase (GPT), glutamic-oxaloacetic transaminase (GOT), troponin (TnI), brain natriuretic peptide (BNP), thyroid hormones (TSH, fT3, fT4).

Thyroid function tests were considered normal according to the range values of the hospital laboratory: TSH 0.3–5 mU/L; fT3 3.9–6.8 pmol/L; fT4 8–20 pmol/L.

A complete cardiological evaluation including Electrocardiogram (EKG) and echocardiography was performed upon admission. In a group of patients, a second dosage of TSH, fT3 and fT4 levels was performed 4 weeks after the onset of the disease.

### Statistical analysis

The continuous variables were collected by calculating the main indicators of centrality and variability. The analysis of the differences in demographic and clinical features and in laboratory values between groups of subjects defined according to variables of interest was made by applying the non-parametric Mann-Whitney *U*-test, after verifying the non normality of the distributions of the variables under examination.

Fisher's exact test or the *χ*^2^ test were used for group comparison (ESS group vs. no ESS group). Spearman's Rho correlation coefficient was calculated to evaluate the correlation between the different laboratory values. A *p*-value less than 0.05 (two-tailed test) was considered statistically significant.

Post-hoc power analysis was calculated on the fT3 values variable (two-tailed hypothesis) given the observed probability level (0.05), the observed effect size (Hedges'g = 2.4202) and the total sample size. Hedges’g was chosen because more appropriate where very different sample sizes are considered, as in the present study. All analyses was performed using IBM SPSS statistical software. (Vers. 20.0).

## Results

### Demographic, clinical and laboratory features of MIS-C patients at onset

In the study we included 42 consecutive children with MIS-C (29 male—69%) with median age 9 years (range 0.7–17 years). As showed in Table 1 the most represented ethnicity was Caucasian (88%), although children of African descent appeared more often involved (9.5%) compared to other ethnic origin. Only 4 subjects had comorbidities [1 periodic fever with periodic fever, aphtous stomatitis, pharyngitis, adenitis (PFAPA syndrome), 2 glucose 6-phosphate dehydrogenase deficiency, 1 autism spectrum disorder]. According to case definition of MIS-C, all patients were exposed to SARS-CoV2. Thirty patients (71%) reported previous confirmed infection with a median time of 4 weeks (range 2–6 weeks).

**Table 1:** Laboratory tests of whole MIS-C group and comparison between patients with ESS and patients with normal thyroid function tests (no ESS group) at the onset of disease.

Variable	Overall (*n* = 42) Median (range)	ESS (*n* = 36) Median (range)	No ESS (*n* = 6) Median (range)	*p* value
White Blood Cells (×10^9^/L)	8.26 (3.03–27.68)	8.53 (3.03–27.68)	7.94 (4.53–12.90)	0.49
Lymphocytes (×10^9^/L)	1.40 (0.41–3.30)	1.43 (0.42–3.30)	935 (0.41–3.04)	0.38
Hemoglobin (g/dl)	11.4 (7.4–13.9)	11.4 (7.4–13.9)	12.3 (10.3–13.7)	0.25
Platelets (×10^9^/L)	159.00 (52.00–449.00)	155.50 (52.00–449.00)	255.50 (127.00–434.00)	**0** **.** **03**
CRP (mg/L)	137 (19.1–546)	163 (47–546)	111 (19.1–152)	**0** **.** **04**
GOT (U/L)	35 (21–221)	35 (21–221)	63 (30–137)	0.24
GPT (U/L)	27 (12–97)	28 (13–97)	28 (12–57)	0.32
Ferritin (ug/L)	550 (121–2,905)	738 (159–2,905)	294 (121–416)	**0** **.** **02**
Troponin I (ng/L)	48 (1.9–29,691)	48 (1.9–1,484)	219 (1.9–29,691)	0.83
Brain natriuretic peptide (ng/L)	202 (8.9–3,731)	211 (9.0–3,731)	82 (9.0–344)	0.14
IL-1α (ng/L)	1.9 (1.9–170)	1.9 (2.0–170)	1.9 (1.9–116)	0.94
IL-1β (ng/L)	7 (3.7–47)	7.2 (4.0–48)	5.6 (3.7–46.5)	0.47
IL-6 (ng/L)	32 (0.5–680)	30 (1.0–680)	79 (9.9–608)	0.26
TNFα (ng/L)	21 (6–75)	21,6 (11–70)	12 (6.3–75.7)	0.10
IL-2r (kU/L)	3,942 (1,190–16,500)	4,452 (1,634–16,500)	1,558 (1,190–2,519)	**0** **.** **00**
S-100 protein (ug/L)	0.27 (0.03–1.83)	0.29 (0.05–1.83)	0.14 (0.03–0.39)	**0** **.** **02**
TSH (mU/L)	1.58 (0.18–4.37)	1.50 (0.18–3.7)	2.70 (1.22–4.37)	0.06
fT3 (pmol/L)	2.55 (0.96–5.36)	2.39 (1.0–3.9)	4.20 (3.94–5.36)	**0** **.** **00**
fT4 (pmol/L)	15.5 (7.3–20.7)	14.7 (7.3–20.7)	17.6 (13.3–18.1)	**0** **.** **05**

Bold values are statistically significant.

Forty-one patients (98%) were treated with immunosuppressive therapy using high-dose immunoglobulin (2 g/kg) plus corticosteroid (methylprednisolone 2 mg/kg). Only one subject was treated only with corticosteroid monotherapy. Due to the severity of the disease onset, six subjects (14%) required treatment with interleukin 1 antagonist (Anakinra). The median time between onset of symptoms and initiation of therapy was 6 days (range 2–9 days).

In Supplementary Table S1, clinical manifestation at onset are reported. Gastrointestinal tract involvement was the most frequent, affecting 39 patients (93%): reported symptoms were mainly abdominal pain (67%), vomiting (45%) or diarrhea (40%). The involvement of cardiovascular system was observed in 34 patients (81%) presenting with severe arterial hypotension in 14 patients (33%), increased myocardial enzymes in 23 patients (54%), decreased cardiac contractility (Ejection fraction EF < 55%) in 16 (38%), or coronary artery abnormalities (coronary ectasias/aneurysms, coronaritis) in 7 (16%). Sixty-seven percent of individuals presented skin and mucosal involvement. Renal and Central Nervous System (CNS) involvement appeared less frequent, involving only 8 (19%). and 4 children (9%), respectively.

No statistically significant difference was found in clinical features between the ESS and no ESS group (Supplementary Table S1).

As shown in Table 1, on admission, 36 children (86%) presented ESS characterized by low fT3 levels (median 2.54 pmol/L, range 0.96–5.36): only one subject presented simultaneously low fT4 levels and another had reduced TSH levels. The post-hoc power analysis showed a very high value (0.9999) although the samples size (ESS group and no-ESS group) were very different.

Other relevant laboratory tests showed increased acute phase reactants such as CRP median 137 mg/L (range 19.1–546) and ferritin median 550 ug/L (range 121–2,905) associated with widely variable range of myocardial enzymes alterations as troponin median 48 ng/L (range 1.9–29,691) and BNP median 202 ng/L (range 8.9–3,731).

At disease onset patients in the ESS group showed a higher inflammatory profile compared with euthyroid subjects. In fact, as showed in Table 1, a statistically significant elevation of the main inflammatory markers such as CRP (*p* = 0.03), ferritin (*p* *= *0.02), IL-2r (*p* *= *0.003), and S-100 protein (*p* *= *0.02) was observed in ESS patients compared with euthyroid ones. The difference in terms of platelet count (*p = *0.03) also appeared significant.

In 16 subjects (2 of them admitted in ICU) the measurement of thyroid function tests was repeated 4 weeks after admission and showed normalization in all (TSH mean 2.57 mU/L, range 0.96–5.36 mU/L, fT3 mean 6,07 pmol/L range 5,2–6,7 pmol/L, fT4 mean 19,2 pmol/L range 15,1–20,1 pmol/L.

### ESS and risk of intensive care support

**Figure 1:**
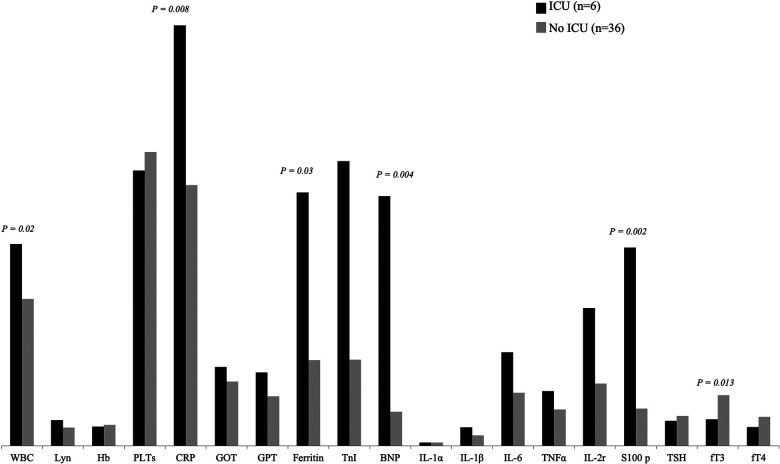
Comparison of laboratory features between patients requiring admission to ICU and patients admitted to general hospital ward.

Among MIS-C patients, 6/42 (14%) required admission to intensive care unit (ICU): these subjects documented much lower fT3 levels compared to other patients (median value 1.45 pmol/L vs. 2.76 pmol/L, *p = *0.013), while no significant difference was observed for fT4 and TSH levels (Figure 1). Indeed, the subjects admitted to ICU, presented significantly higher inflammatory markers and white blood cells count: respectively WBC (median value 11,355/mmq vs. 8,060/mmq, *p = *0.019), CRP (median value 229 mg/L vs. 143 mg/L, *p = *0.008), ferritin (median value 1,383 ug/L vs. 467 ug/L, *p = *0.034), BNP (median value 1,363 ng/L vs. 187 ng/L *p = *0.004) and S100 protein (median value 1.08 ug/L vs. 0.24 ug/L, *p = *0.002).

### ESS and risk of severe cardiac involvement

**Figure 2:**
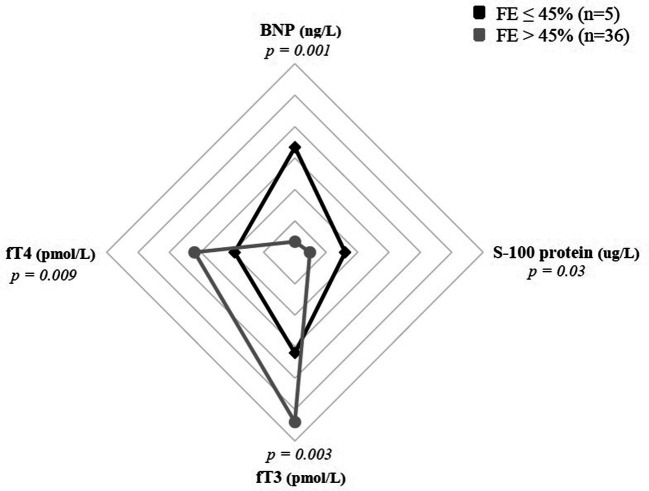
Correlation between severity of myocardial dysfunction and ESS.

Severe depression of myocardial function, defined by EF ≤ 45%, was found in 5 patients (12%) of MIS-C cohort. This subgroup presented significantly lower fT3 values (median 1.55 pmol/L vs. 2.73 pmol/L; *p* *= *0.003) and fT4 (median 9.64 pmol/L vs. 16.02 pmol/L; *p = *0.009) if compared with EF > 45% subgroup, as showed in Figure 2. Conversely, no clear association between fT3 values and presence of coronary dilatation at the onset of the disease has been detected.

### Prognostic role of ESS for severe clinical course

**Figure 3:**
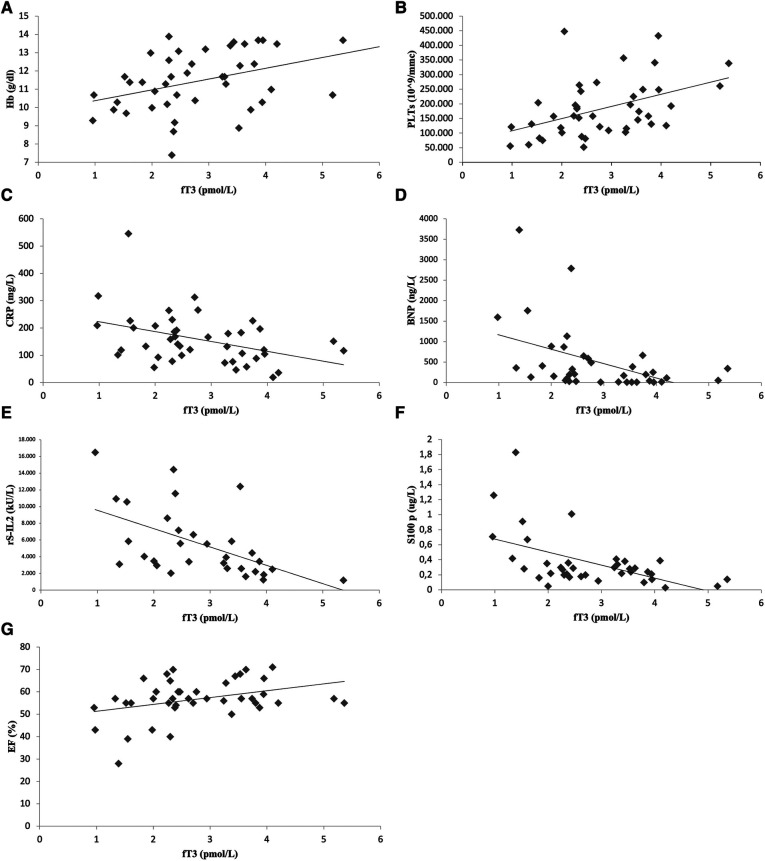
Significant correlation between laboratory-instrumental values ​​and fT3 level: scatterplots show correlation between fT3 and hemoglobin (**A**), platelets (**B**), C-reactive protein (**C**), brain natriuretic peptide (**D**), soluble IL-2 receptor (**E**), S -100 protein (**F**) and ejection fraction (**G**).

As shown in Figure 3A,B, statistical analysis demonstrated a direct correlation between ESS severity, expressed by fT3 values, and hemoglobin levels [correlation coefficient (*ρ*) 0.39, *p = *0.01] or platelet count (*ρ* 0.44, *p = *0.003). An inverse correlation was documented between fT3 levels and markers of systemic inflammation such as CRP (*ρ* 0.390, *p = *0.009), soluble IL2 receptor (*ρ* 0.59, *p = *0.001) and S-100 protein (*ρ* 0.39, *p = *0.002) (Figure 3C,E,F). Moreover, a statistically significant inverse correlation was observed between triiodothyronine and BNP (*ρ* 0.49, *p* = 0.002) (panel D, Figure 3). About instrumental data, there was a direct correlation between fT3 levels and EF values on admission (*ρ* 0.36, *p *= 0.017), as shown in panel F of Figure 3. No statistically significant correlation between fT3 values and fever duration at the time of initiation of therapy, WBC (*ρ* −0.26, *p = *0.09), lymphocytes count (*ρ* −0.16, *p = *0.33), GPT (*ρ* −0.16, *p = *0.3), GOT (*ρ* 0.10, *p = *0.5), ferritin (*ρ* −0.15, *p = *0.39), IL-1*α* (*ρ* −0.18, *p = *0.26), IL-1β (*ρ* −0.26, *p* = 0.11), IL-6 (*ρ* 0.12, *p = *0.45), TNF (*ρ* −0.33, *p = *0.055), TnI levels (*ρ* −0.03, *p = *0.82).

## Discussion

During the COVID19 pandemic, thyroid function alterations have been reported in patients not previously diagnosed with any thyroid conditions. The mechanisms underlying COVID-19-related dysfunction of endocrine glands consist of inflammation, vessel damage, necrosis, degeneration, immune and autoimmune processes (11–14). For the abnormalities of the hypothalamus-pituitary-thyroid axis associated with COVID19 several mechanisms have been suggested such as a disturbance in the TSH process via virus-related hypophysitis, or a subacute thyroiditis linked to the virus spread (11, 15). Subacute thyroiditis during COVID19 presents with thyroid hormone flare-up, usually is self-limited and does not require specific treatment (16, 17). Two major pathophysiological models have been implicated: a direct infection of thyroid cells by SARS-CoV-2 as they express Angiotensin-converting enzyme (ACE) 2 or an indirect effect caused by an immune-inflammatory abnormal response to the virus, especially at the moment of cytokine storm, in addition to multiple organ failure (18–21).

In patients with COVID19 other thyroid function parameters alterations, which are commonly referred as euthyroid sick syndrome (ESS) have been reported (22). ESS has been recognized since the 1970s when it was observed that acute illnesses and fasting may affect circulating levels of thyroid hormones in subjects without previously diagnosed thyroid disease. Most typically, in ESS plasma concentrations of fT3 decrease and those of rT3, the biologically inactive form of fT4, rise (23, 24). This suggests an inactivation of thyroid hormone in peripheral tissues likely mediated by activation of type 3 deiodinase (D3) or by suppressed activity of type 1 deiodinase (D1) (25, 26). As the severity and the length of ESS increases, also the fT4 levels can be deeply reduced (27).

ESS has been reported both in adult and pediatric populations in many severe clinical conditions such as sepsis, trauma, acute myocardial infarction, severe malnutrition, liver failure, cardiac surgery, and diabetic ketoacidosis (5–9). Zou et al. studied 149 COVID19 patients and found that those with ESS (27.5%) had more severe inflammatory responses, such as higher levels of C-reactive protein and erythrocyte sedimentation rate compared to those without ESS (22). In a cohort of subjects with acute COVID19 infection, the presence of ESS correlated with a more severe course of the disease, with higher inflammatory parameters level and risk of myocardial dysfunction compared with normal thyroid profile (28).

In pediatric age, beside a low rate of hospitalization and need for intensive support associated with direct SARS-CoV2 infection, a great burden of morbidity was observed because of the novel multisystem inflammatory syndrome in children (MIS-C) related to COVID19 (1, 2).

MIS-C is an immune-mediated disease that can manifest with multiple organ failure and even lead to shock. The etiology of this syndrome is not yet fully understood: host genetic factors associated to alterations of innate and adaptive immune system as well as mechanisms of molecular mimicry (29) can develop MIS-C as a result of a cytokine storm after the infection (30, 31).

The clinical features and the involvement of internal organs in MIS-C have been extensively studied while only few data are available on dysfunction of endocrine systems and its possible clinical relevance. Calcaterra et al. documented that 23/26 patients (88%) with MIS-C presented ESS at onset predominantly characterized by low fT3 values (65%) as compared to alterations of other thyroid hormones (4). Similarly, in a case-control study, lower fT3 levels were found in patients with MIS-C compared with healthy subjects with opposite results for fT4 levels (32).

In the present study, we assessed the thyroid function in patients with MIS-C and analyzed the potential value of ESS in predicting disease severity. In our series, similarly to previous studies, ESS was present in the majority of patients (86%) with an hormonal profile mainly characterized by reduced fT3 values. In our cohort we did not find statistically significant differences in clinical features between patients with ESS and those with normal thyroid levels, probably due to the small number of patients. Nevertheless, it is worth to note that signs of more severe course such as hypotension, coronary abnormalities, CNS and renal involvement were more frequent in patients with ESS, and this was confirmed by the observation that children requiring admission in intensive care unit were all from the ESS group.

Furthermore, our patients with ESS showed markedly more elevated inflammatory markers such as CRP, ferritin, rsIL-2 and S-100 protein and we observed a strong correlation between fT3 levels and different inflammatory markers while they did not correlate with the duration of the disease. These results suggest that the hyperinflammatory state could be principal responsible for the hormone dysfunction and related to its severity.

This evidence suggests ESS as a useful adaptation of the body to counteract excessive catabolism during illness and as a part of the acute phase response mediated by cytokines (33). In fact, proinflammatory cytokines, especially IL-1, IL-6, TNF-α, and interferon-γ, inhibit several genes involved in thyroid hormone metabolism *in vitro* (34, 35). The administration of cytokines in experimental models resulted in altered thyroid hormone metabolism exhibiting some, but not all, features of disease-related ESS. A possible causal role for IL-6 in the development of ESS was suggested since IL-6 knock out mice showed a less pronounced drop in serum T3 during illness (36). However, acute injection of cytokines induced a flu-like illness but failed to induce ESS like features, except for interferon gamma (IFN-γ) which reduced serum T3 and T4, therefore suggesting that the resulting illness, rather than the cytokines alone, accounted for the changes in thyroid hormones metabolism (37). Other studies showed that several components of the thyroid hormones synthesis pathway can be downregulated by cytokines directly on the level of the thyrocyte, ultimately leading to decreased secretion of T4 and T3 (38). Moreover, studies *in vitro* and in animal models showed that IL-1α and IL-1b may display multiple effect such as inhibition of the thyroglobulin (Tg) mRNA expression induced by TSH and subsequent Tg release in human cultured thyrocytes and decrease of ^125^I incorporation and T4 and T3 secretion from human thyrocytes (34, 39, 40). TNF*α* plays an important role in the acute phase response and *in vitro* studies showed that it inhibits the TSH-induced cAMP response and thyroglobulin production and release in cultured thyrocytes (39, 41, 42).

Some evidence shows that pro-inflammatory cytokines may contribute to development of ESS also by affecting the expression and the activity levels of the deiodinases. In particular, during inflammation, competition for co-factors by cytokine-induced pathways such as nuclear factor-kappa B (NF-kB) are associated with less transcription of the D1 gene in the liver and with increase in D2 expression in the hypothalamus (43–45).

Although with the limitation of a small number of patients our data suggest that both adaptation and cytokines hyperproduction probably act together to development of ESS which entity, in our cohort, directly correlate with severity of clinical picture. Moreover, our data suggest that repeated dosages of fT3 levels may be useful to monitor the disease course.

In agreement with the role of ESS as useful and transient mechanism of adaptation during severe diseases, such as MIS-C, to reduce catabolic processes, in our cohort the levels of thyroid hormones spontaneously turned to normal during the first month from disease onset.

In our study, among the 36 subjects with ESS, only two had alterations of TSH or fT4 levels: these data differ from those reported in literature where the rate of ESS with an isolated reduction of fT3 was observed in only 65% of patients (4). In our patients diagnosis and treatment were started quite early after disease onset, therefore this could suggest a greater peripheral block of conversion of fT3 than of fT4 and a lack of central inhibition of TSH in our cohort.

In our study patients who needed admission to ICU presented lower levels of fT3 and moreover we showed a strong direct correlation between fT3 levels and severity of myocardial depression on admission. In fact we retrieved that suppression of fT3 was associated with EF < 45% and inversely correlated with BNP levels.

The relationship between ESS and heart dysfunction has been extensively investigated. Low serum fT3 concentrations are a negative prognostic factor in patients with congestive heart failure, raising a question whether thyroid hormones may play a role in acute cardiac injury (46, 47). In children undergoing cardiac surgery more severe ESS changes are associated with prolonged hospital stays and increased ICU and mechanical ventilation requirements (48, 49).

A possible explanation of this relationship is that in cardiomyocytes, deiodinase D3 expression is low under physiological conditions while its activity is upregulated in case of myocardial infarction (50, 51). Another possible contributing factor is hypoxia because increased level of peripheral D3 are induced by hypoxemia due to decreased tissue perfusion during illness (52, 53).

Our study has some limitation due to the small number of patients secondary to the low prevalence of MIS-C and the monocentric nature of the study. Despite the very different samples size the post-hoc power analysis was very good, thus confirming the statistical significance of our findings. Indeed, further studies on larger populations should be needed to confirm the data.

## Conclusion

Our study showed that most of MIS-C patients has ESS, mainly characterized by low levels of fT3. In addiction, results suggest that ESS, particularly fT3 level, is an independent risk factor for the disease severity of MIS-C. In fact, patients with ESS and lower fT3 had stronger inflammatory responses, more severe cardiac involvement and higher risk of ICU requirement. These findings may be helpful in clinical practice because fT3 is processed by conventional laboratory in urgency, therefore it could be used as an additional data in prompt decision making in patients with MIS-C.

## Data Availability

The original contributions presented in the study are included in the article/**Supplementary Material**, further inquiries can be directed to the corresponding author.
