# Supplementary material for: Prognostic role of euthyroid sick syndrome in MIS-C: results from a single-center observational study
Source: Front Pediatr. 2023 Aug 10;11:1217151. doi: 10.3389/fped.2023.1217151 (PMC10448823; doi:10.3389/fped.2023.1217151)
Supplement: Supplementary file 1 [file Table1.pdf]

Table 1. Clinical characteristics of the whole cohort of MIS-C patients and of the groups of patients with ESS and with normal thyroid function tests (no ESS)

**Variable**	**Overall - n.42(%)**	**ESS - n.36 (%)**	**No ESS - n.6 (%)**	***p value***
**Age (median)**	9 years (0.7 – 17.7)	9 years (0.7 – 17.7)	8 years (2.2 – 13.9)	0.774
**Sex no. (%)**				
Male	29 (69)	26 (72)	3 (50)	0.353
**Race no. (%)**				
Caucasian	37 (88)	32 (89)	5 (83)	0.557
African	4 (9)	3 (8)	1 (16)	0.474
Other	1 (1)	1 (3)		> 0.999
**Comorbidity no. (%)**	4 (9)	2 (4)	2 (33)	0.091
**Clinical features no. (%)**				
**Gastrointestinal**	39 (92)	33 (92)	6 (100)	> 0.999
Abdominal pain	28 (67)	23 (64)	5 (83)	0.645
Vomit	19 (45)	18 (50)	1 (16)	0.197
Diarrhea	17 (40)	14 (39)	3 (50)	0.672
**Cardiovascular**	34 (81)	30 (83)	4 (66)	0.319
Hypotension	14 (33)	13 (36)	1 (16)	0.645
↑ Troponin	23 (55)	20 (55)	3 (50)	> 0.999
↑ BNP	25 (59)	23 (64)	2 (33)	0.202
Coronary involvement	7 (17)	7 (19)	0 (0)	0.567
EF ≤55	16 (38)	14 (39)	2 (33)	> 0.999
**Skin**	28 (67)	25 (69)	3 (50)	0.383
**Renal**	8 (19)	8 (22)	0 (0)	0.576
**Central Nervous System**	4 (9)	4 (11)	0 (0)	> 0.999
**ICU admission no. (%)**	6 (14)	6 (17)	0 (0)	0.569

p value is significant when <0.05
